# A Route to Understanding the Ethane Adsorption Selectivity of the Zeolitic Imidazolate Framework-8 in Ethane–Ethylene Mixtures

**DOI:** 10.3390/ma16196587

**Published:** 2023-10-07

**Authors:** Jaquebet Vargas-Bustamante, Roberto Salcedo, Jorge Balmaseda

**Affiliations:** Departamento de Polímeros, Instituto de Investigaciones en Materiales, Universidad Nacional Autónoma de México, Circuito Exterior s/n, Ciudad Universitaria, Coyoacán, Ciudad de México 04510, Mexico; jaquebet@comunidad.unam.mx (J.V.-B.); balmaseda@comunidad.unam.mx (J.B.)

**Keywords:** ZIF-8, ethane, ethylene, selectivity, hydrogen bond

## Abstract

Ethylene production has a negative environmental impact, with its separation step being one of the major contributors of pollution. This has encouraged the search for energy-efficient alternatives, among which the adsorptive separation of ethane and ethylene stands out. ZIF-8 is a molecular sieve that is potentially useful for this purpose. It is selective to ethane, an exceptional property that remains unexplained. Furthermore, the adsorption of ethane and ethylene above room temperature, such as at steam cracking process outlet temperatures, has not been addressed either. This work aims to fill this knowledge gap by combining experiments at very low volumetric fillings with density–functional theory modelling methods. Adsorption isotherms of ethane and ethylene on ZIF-8 at pressures below 0.3 bar and 311 K, 333 K, and 363 K were measured using zero-length column chromatography. The low-pressure domain of the isotherms contains information on the interactions between the adsorbate molecules and the adsorbent. This favors the understanding of their macroscopic behavior from simulations at the atomic level. The isosteric enthalpy of adsorption of ethane remained constant at approximately −10 kJ/mol. In contrast, the isosteric enthalpy of adsorption of ethylene decreased from −4 kJ/mol to values akin to those of ethane as temperature increased. ZIF-8 selectivity to ethane, estimated from ideal adsorbed solution theory, decreased from 2.8 to 2.0 with increasing pressure up to 0.19 bar. Quantum mechanical modelling suggested that ethylene had minimal interactions with ZIF-8, while ethane formed hydrogen bonds with nitrogen atoms within its structure. The findings of this research are a platform for designing new systems for the adsorptive separation of ethane and ethylene and thus, reducing the environmental impact of ethylene production.

## 1. Introduction

Ethane and ethylene are two gases that coexist within the mixture of various substances released during the extraction of crude oil from oil wells. To purify these gases, subsequent petrochemical processes are employed. One such process is cryogenic distillation, unfortunately characterized by its substantial energy consumption, thereby becoming a significant source of CO_2_ emissions [1,2]. This heightened energy consumption has prompted an intensified quest for more efficient and environmentally friendly alternatives [3]. In this context, adsorption separation, based on the utilization of porous solid materials, has emerged as a promising alternative due to its cost-effectiveness and reduced energy consumption [4].

Metal–organic frameworks, commonly referred to as MOFs, have garnered increasing attention in recent times due to their remarkable properties that find applications in catalytic, adsorption, and charge transfer processes [5,6]. Among the various outstanding features of MOFs, their exceptional gas adsorption capacity stands out prominently [5,6]. Specifically, the zeolitic imidazolate-8 framework (ZIF-8) has gained widespread recognition for its outstanding adsorption capabilities. What makes ZIF-8 unique is its ability to amalgamate the inherent advantages of MOFs with the structure of a natural zeolite. Particular emphasis has been placed on ZIF-8’s capacity for the separation of olefins and paraffins, especially concerning ethane and ethylene [7].

Most of the previous studies concerning the separation of ethane and ethylene within the zeolitic imidazolate-8 (ZIF-8) structure have emphasized its pronounced selectivity toward ethane within specific pressure regimes, as substantiated by references [8,9]. Nonetheless, it is crucial to acknowledge that gas mixtures originating from steam cracking furnaces typically exhibit temperatures above ambient levels. Consequently, it becomes imperative to examine the adsorbent’s performance under conditions that more accurately mirror the realities of industrial settings.

In an alternate context, the exploration of the low-pressure region within adsorption isotherms yields valuable insights into the interactions between the adsorbate and the adsorbent [10]. These insights can prove instrumental in refining separation procedures. Importantly, it should be noted that, as of now, there are no prior studies addressing temperatures exceeding 298 K while concurrently analyzing the low-pressure segment of the adsorption isotherms for ethane and ethylene on ZIF-8.

The zero-length column chromatography (ZLC) technique [11] presents distinct advantages for the investigation of porous solid adsorbents due to its simplicity and cost-effectiveness, surpassing other techniques in these aspects. Through the utilization of an exceedingly short and densely packed column, diffusion and mass transfer effects are significantly reduced, thereby enabling a more comprehensive and precise analysis of the interactions that occur within the low-pressure region of the adsorption isotherm [12].

In the present study, adsorption isotherms of ethane and ethylene on a ZIF-8 structure were determined at three different temperatures using ZLC technique. Based on this experimental data, selectivities as a function of temperature were calculated by employing the ideal adsorbed solution theory (IAST). Additionally, interactions between the adsorbate and the adsorbent were explored through electronic structure modeling of the structures. This analysis was conducted with the aim of comprehending how Zn–N coordination and the presence of nitrogen on the periphery of the ZIF-8 structure positively influenced ethane adsorption and selectivity processes.

## 2. Materials and Methods

### 2.1. Synthesis of ZIF-8 in Methanol

The compound ZIF-8 was synthesized starting from zinc nitrate hexahydrate [Zn(NO_3_)_2_·6H_2_O, Sigma-Aldrich (Saint Louis, MO, USA), 99%], methanol [MeOH, Interchema, Pract.], and 2-methylimidazole [HMeIm, Acros Organics, Geel, Belgium, 99%]. The Zn(NO_3_)_2_·6H_2_O (0.21 g, 7.06·10^−4^ mol) was dissolved in 10 mL of methanol and, in turn, 2-MeIm (0.46 g, 8.04·10^−3^ mol) was dissolved separately in 10 mL of methanol. Both solutions were then mixed in a 20 mL beaker and shaken vigorously for 30 s. The resulting mixture was kept at room temperature for 24 h. The crystals were obtained by centrifuging twice for 5 min at 4000 rpm and washed with 20 mL of methanol. Subsequently, the obtained crystals were dried at room temperature for one day and then for 12 h in an oven at 348 K with a heating ramp of 278 K/min [13].

X-ray diffraction, thermogravimetric analysis, infrared spectroscopy, and scanning electron microscopy were used to characterize the ZIF-8 product.

### 2.2. Characterization

Powder X-ray diffraction patterns were recorded in a Siemens D5000 diffractometer with Kα irradiation of Cu. The diffraction measurements on the different solids were conducted under uncontrolled environmental conditions (around 0.77 bar and 294 K). All the samples were measured between 5° and 65° in 2θ with an angular step of 0.02° and a counting time of 2 s for each step. Prior to the recording of powder patterns, the samples were pulverized in an agate mortar. The diffraction patterns were analyzed using FullProf/2.05 software.

Thermogravimetric analysis of ZIF-8 was performed on a simultaneous thermoanalyzer SDT Q600 (TA Instruments, New Castle, DE, USA) in an air atmosphere with a heating rate of 10 °C/min from room temperature to 1068 K. Universal Analysis 2000v4.3A software was used to analyze the thermograms.

Fourier-transform infrared (FT-IR) spectra were collected using attenuated total reflection (ATR) sampling on a Thermo Fisher Scientific Nicolet (Waltham, MA, USA) iS5 spectrometer. FT-IR spectra were recorded from 400 cm^−1^ to 4000 cm^−1^, at a resolution of 4 cm^−1^, with an average of 32 scans. Thermo Fisher Scientific OMNIC™ spectroscopy software, version 4.5A, was used to analyze the data.

Morphology and particle size distribution (PSD) were studied with a Schottky JSM-7800F field emission scanning electron microscope from Jeol. Crystal sizes were measured using ImageJ 1.53t software [14]. The average particle size and standard deviations were determined from the Gaussian fit. The adsorbent particles were assumed to be uniform microporous spheres with a diameter equal to the mean particle size in ZLC mass balance.

### 2.3. Adsorption Experiment and Analysis

Ethane and ethylene adsorption experiments were performed at equilibrium conditions using the semi-automated ZLC system described in a previous report [11]. ZIF-8 was degassed for 12 h at 480 K with a helium 6.0 (Praxair, Danbury, CT, USA) flow rate of 5 cm^3^/min, in agreement with the temperature at which the desolvation (dehydration) event ended on the thermogravimetric curve. The adsorbent was saturated with a feed comprising 10% diluted ethane or ethylene, along with a carrier gas (helium 6.0 Praxair), for 1 h at three different constant temperatures: 311 K, 333 K, and 363 K. In order to ensure accurate and representative measurements, careful manipulation of the adsorbate concentration on the ZLC column was carried out. To achieve this, pre-tests were performed with different concentrations, thus establishing a range of optimal values. This ensured that all measurements were performed at low concentrations, ensuring that they remained within the equilibrium regime, consistent with ZLC theory [15]. Once the sample reached saturation, the desorption process was performed with a pure helium stream and the desorption curve was recorded.

Analysis of adsorption data was performed in pyGAPS 4.5.0 suite [16]. The Clausius–Clapeyron method implemented in the suite was used to calculate the molar enthalpy of the adsorption curve. Selectivities were calculated under the assumptions of IAST and using linear interpolations.

### 2.4. Computational Methods

All the molecules were optimized by applying a hybrid method based on the combination of Becke’s gradient corrections [17] for exchange and Perdew–Wang’s for correlation [18]. This was the scheme used for the B3PW91 method, which is included in the Gaussian 16 [19] package. Molecular orbitals and spectroscopic studies arose from M06 [20] calculations carried out on optimized structures. This method was chosen because it has demonstrated high accuracy for this kind of result [21]. The calculations were performed using the 6-31G** basis set. To confirm that the optimized structures were at a minimum on the potential energy surface, frequency calculations were carried out at the same level of the theory used for the geometrical calculation.

## 3. Results and Discussion

### 3.1. Material Characterization

**Figure 1:**
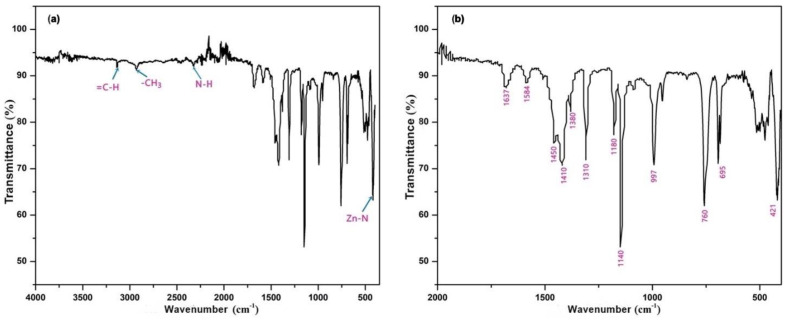
Fourier−transform infrared (FT-IR) spectra of (**a**) the 4000–460 cm^−1^ region and (**b**) the 1850–500 cm^−1^ region of synthesized ZIF-8.

The molecular formula of ZIF-8 ($\mathrm{Zn}{\left(C_{3}H_{3}N_{2}\right)}_{2}$, or in its simplified form, Zn(Im)_2_), is composed of zinc cations and imidazolate $\left(C_{3}H_{3}N_{2}^{-}\right)$ anions. In the ZIF-8 framework, the zinc ions bind to the imidazole anions to form a three-dimensional network. All functional groups present in the crystal chemical formula of ZIF-8 were identified by their FTIR spectra (Figure 1). The bands observed in the spectra were consistent with the expected vibrations of the imidazole ring and MOF structure. The presence of the stretching bands at 3135 cm^−1^ and 2929 cm^−1^ indicates the presence of aromatic C–H and aliphatic C–H groups, respectively. These bands are characteristic of the imidazole ring, which is a key building block of the ZIF-8 framework.

The peak at 2222 cm^−1^ corresponds to the NH stretching vibration present in imidazole rings [22]. This peak is an important indicator of the presence of imidazole rings, which are essential components of ZIF-8. The hydroxyl bending band at 1637 cm^−1^ further supports the presence of coordinated hydroxyl groups, which are known to be involved in the formation of MOFs through coordination with metal cations. The peak at 1584 cm^−1^ is assigned to the C=N stretching mode, which is another important characteristic of the imidazole ring.

The spectral region between 600 cm^−1^ and 1500 cm^−1^ is associated with full ring stretching or bending, which is another indication of the presence of the imidazole ring in the ZIF-8 framework. Finally, the band observed at 421 cm^−1^ corresponds to the stretching of Zn–N, which further confirms the coordination of the imidazole linkers with the Zn cations [23,24,25].

**Figure 2:**
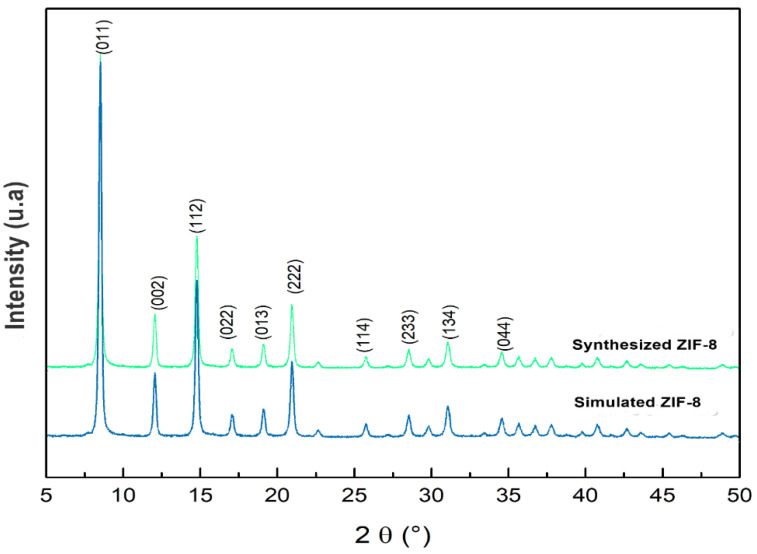
Powder diffraction patterns of synthesized and simulated ZIF-8.

The XRD powder pattern of the synthesized sample closely matched the simulated pattern of SOD-type ZIF-8 (Figure 2). According to the powder pattern, the reflections at 2θ = 7.30°, 10.35°, 12.70°, 14.90°, 16.40°, 18.00°, 24.9°, 25.5°, and 26.6° correspond to the planes (011), (002), (112), (022), (013), and (222), respectively [26,27]. This result confirms that the sample was a pure phase of ZIF-8 [28,29,30,31].

**Figure 3:**
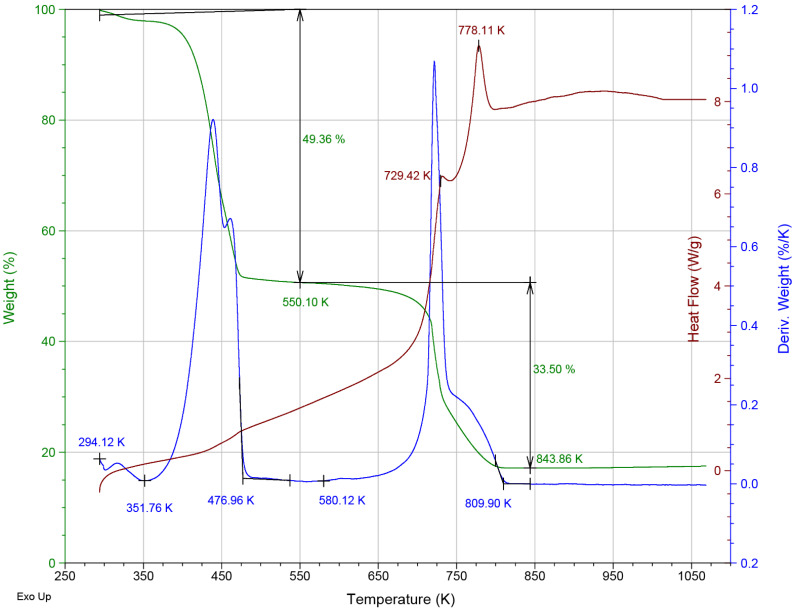
Thermogravimetric (green), derivative thermogravimetric (blue), and calorimetric (maroon) curves for ZIF−8.

The derivative thermogravimetric curve of ZIF-8 exhibits five distinct thermal events, as depicted in Figure 3. These events are characteristic of ZIF-8 and have been well documented in previous studies [23,32,33,34]. The initial three events, spanning from room temperature to approximately 550 K, collectively resulted in a weight loss of 49% (as shown in Figure 3) and could be attributed to the desorption of adsorbed species from the surface and the desolvation of the ZIF-8 framework.

The event concluding at 352 K is likely linked to surface desorption, while those occurring between 350 K and 550 K are associated with the release of water and ethanol from the framework cavities (as illustrated in Figure 3). Given the structural similarities of these molecules, it is challenging to discern these events separately. It is important to note that water and ethanol molecules were bound to the lattice of the material via hydrogen bonds. The presence of OH groups in the adsorbed water molecules is corroborated by the broad absorption band observed in the infrared spectrum from 3000 cm^−1^ to 3600 cm^−1^ (as demonstrated in Figure 1).

At 550 K, the thermal decomposition of ZIF-8 was initiated through a multi-step process (Figure 3). This multi-step decomposition led to the presence of two overlapping peaks in the DTG curve spanning from 550 K to 810 K. The total weight loss associated with this decomposition process amounted to 33.5%. It is important to note that this weight loss is attributable to the release of volatile compounds and gases generated during decomposition [32].

In the DSC thermogram, two distinct exothermic peaks can be observed at 729 K and 778 K (as depicted in Figure 3). These processes can be attributed to the combustion of imidazolate ligands, which aligns with the observed weight loss phenomenon.

**Figure 4:**
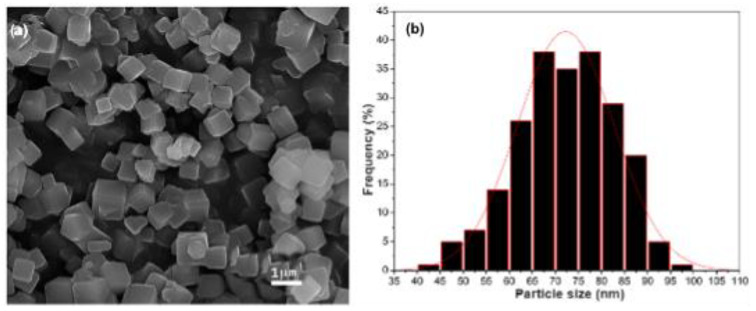
SEM micrograph of (**a**) ZIF-8 powder and its (**b**) particle size distribution.

The ZIF-8 SEM micrograph (Figure 4a) shows cubic crystals with a uniform morphology. Larger polycrystalline particles also appear in the micrograph. However, no crystals other than cubic crystals were observed. Particle size distribution (Figure 4b) could be described by a log-normal distribution with a mean at approximately 75 μm. The morphology described above agrees with a previous report [23], which supports the validity of the synthesis method employed. It should be noted that the average particle size is large enough to perform ZLC experiments. Because adsorption takes place on the surface of the particles, their larger surface area implies a larger number of available adsorption sites. When conducting adsorption experiments, it is essential to consider this kinetic aspect and adjust the contact time appropriately to ensure that complete equilibrium is reached [35].

### 3.2. Ethane and Ethylene Adsorption

The adsorption isotherms of ethane and ethylene on ZIF-8 at 311 K closely resemble those reported previously for the same adsorbate–adsorbate systems at 298 K [8,9]. It is worth noting that at 298 K, ethylene was above its critical temperature, while ethane was only 7 K below its critical temperature, as indicated in Supplementary Table S1 [36,37,38,39]. Under the pressures employed in the adsorption experiments, as illustrated in Supplementary Figure S2 [7], both components exhibited gas-like behavior. Assuming an ideal gas model and considering that the temperature difference between the reported isotherm and the experimental data in this study did not exceed 4%, we would have expected a comparable change in the adsorbed quantity. Such a change was not significant in the context of an adsorption experiment, which explains the observed similarity between the isotherms at 298 and 311 K.

**Figure 5:**
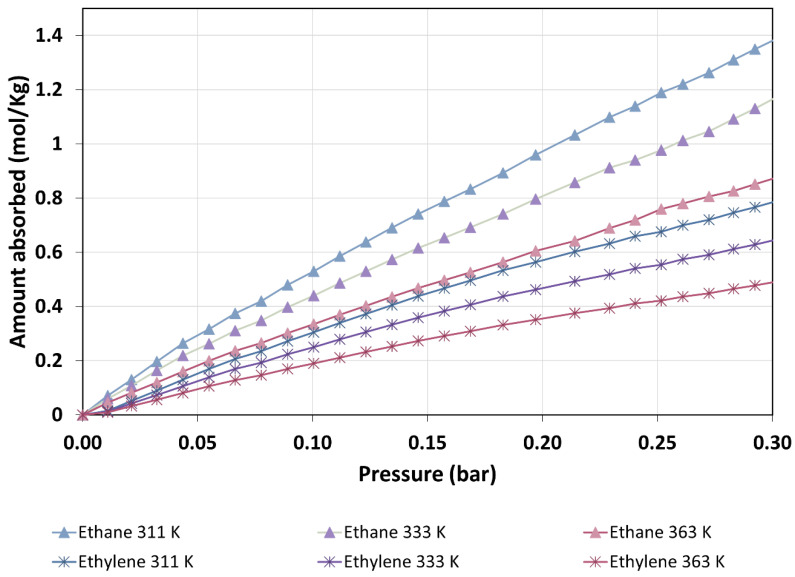
Adsorption isotherms derived from the integration of the ZLC response curves of ethane and ethylene on ZIF-8 at various temperatures.

The quantities of adsorbed ethane consistently surpassed those of ethylene across the entire pressure range studied at three different temperatures (311, 333, and 363 K), as clearly depicted in Figure 5. Additionally, it is important to highlight that the amount of adsorption decreased as temperature increased at a constant pressure for each adsorbate. This phenomenon signifies a significant change in the molar volume of the adsorbed phase with variations in temperature. Such behavior is characteristic of cases where adsorption is non-localized, primarily due to relatively weak interactions between the adsorbate and adsorbent when compared to thermal energy.

**Figure 6:**
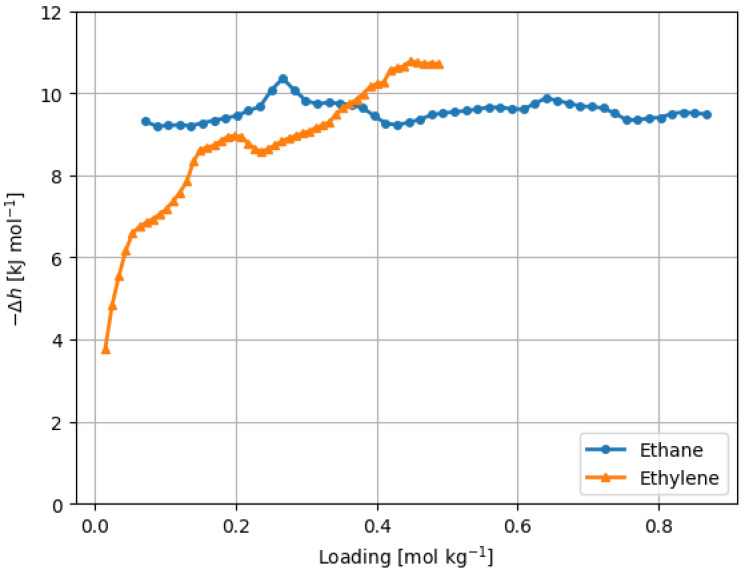
Isosteric enthalpy of adsorption of ethane and ethylene on ZIF-8. The enthalpies were calculated from the Clausius–Clapeyron equation and isotherms at 311 K, 333 K, and 363 K.

The isosteric enthalpy of adsorption represents a pivotal and informative thermodynamic parameter, serving as a powerful tool for the quantitative analysis of adsorbate–adsorbent interactions. Its significance was most pronounced under low-pressure conditions, a regime wherein such interactions took center stage, as clearly demonstrated in Figure 6. Of particular note is the intriguing divergence observed in the isosteric enthalpy of adsorption between ethane and ethylene within this context. Ethane consistently exhibited a more negative isosteric enthalpy in comparison to ethylene across the entire gamut of adsorbed quantities.

For ethane, the isosteric enthalpy maintained remarkable stability, remaining virtually constant at approximately −10 kJ/mol throughout the entire adsorption range. This constancy implies a consistent adsorption mechanism, signifying that the adsorbate–adsorbent interactions were governed by a well-defined energy landscape. This finding holds significant implications for practical applications involving ethane adsorption, such as gas storage or separation processes. Conversely, the isosteric enthalpy of adsorption for ethylene exhibited a contrasting trend. At low adsorbate loadings, it displayed a gradual, monotonically decreasing pattern. This behavior suggests that the initial stages of ethylene adsorption were characterized by relatively stronger interactions between the adsorbate and adsorbent, as evidenced by the decreasing enthalpy. However, as the loading increased and approached 0.4 mol/kg, the isosteric enthalpy leveled off, converging to values approximating −10 kJ/mol (as shown in Figure 6). This plateau indicates a shift toward a more uniform adsorption behavior as the adsorbate quantity increased.

The enthalpy of adsorption, measuring at approximately −10 kJ/mol, closely approximated the vaporization enthalpy values for both molecules (as detailed in Supplementary Table S1 [36,37,38,39]). Such proximate adsorption enthalpy values to vaporization enthalpies indicate relatively feeble adsorbate–adsorbent interactions. The detailed examination of the nature of these interactions will be undertaken in subsequent sections of this study.

ZIF-8, being a heterogeneous adsorbent as documented in previous studies [8,9,29], did not exhibit a consistent isosteric enthalpy of adsorption, as observed with ethane. Such behavior can only be elucidated by considering a compensation effect arising from the interplay between the declining adsorbate–adsorbent interactions and the escalation of adsorbate–adsorbate interactions as the loading increased. In the case of ethylene, it appears that adsorbate–adsorbate interactions took precedence over adsorbate–adsorbent interactions, indicating that ethane interacted more robustly with ZIF-8.

Moreover, despite their similar kinetic diameters (4.443 Å for ethane and 4.163 Å for ethylene [40]), the linear structure of ethane may have facilitated its easier accommodation within the tetrahedral pores of ZIF-8, while the planar geometry of ethylene might have encountered more challenges in fitting within these pores. This geometric consideration could have contributed to a higher affinity of ethane for adsorption on ZIF-8 [41], consequently leading to a greater quantity of ethane being retained compared to ethylene.

While both ethane and ethylene could access the pores of ZIF-8, it has been observed that ethane exhibited a higher adsorption preference compared to ethylene. Although steric hindrance could initially have accounted for this preference, it has been revealed that it was not the sole determining factor. In fact, the preference for ethane arose from disparities in the electrical properties of the gases and their interactions with the adsorbent surface of ZIF-8. Specifically, it has been noted that weak interactions, such as van der Waals forces and hydrogen bonds, exhibit greater strength between the methyl group (–CH_3_) of ethane and the nitrogen linkers (2-MeIm) present in the ZIF-8 framework. In contrast, these interactions are weaker between the methylene group (–CH_2_) of ethylene and the coordinating groups within the ZIF-8 framework, resulting in a lower recovery of ethylene compared to ethane [29].

Now, we will consider ZIF-8 as an adsorbent of ethane/ethylene mixtures. The ideal adsorbed solution theory enabled us to make reasonable predictions regarding mixture adsorption based on the isotherms of the individual components. To illustrate, consider a scenario in which a mixture comprises an equal quantity of both gases, and its pressure remains below 0.175 bar. It is essential to note that this pressure limit is dictated by the experimental conditions (as depicted in Figure 5) and the interpolation method employed for calculations. While it is conceivable to extrapolate this to higher pressures, doing so would introduce uncertainties greater than the uncertainties already associated with the IAST assumptions.

**Figure 7:**
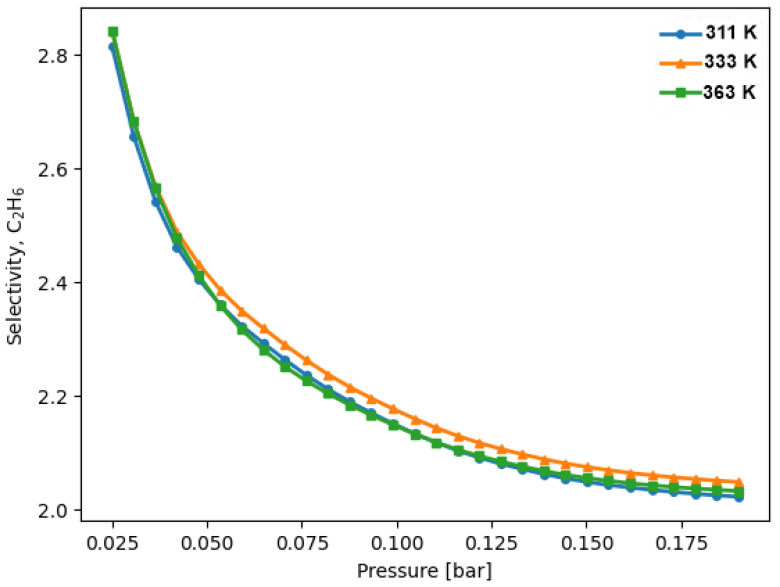
IAST-predicted selectivity for a 0.5:0.5 mixture composition of ethane over ethylene on ZIF-8 at different temperatures.

The selectivity of ZIF-8 for ethane decreased from 2.8 to 2.0 within the pressure range under investigation (Figure 7). This behavior aligns with the evolution of the adsorption enthalpy (Figure 6). With increasing pressure, the disparity between the adsorption enthalpies diminished, resulting in a corresponding decrease in selectivity for ethane. This trend can be attributed to ethane possessing the most negative enthalpy among the adsorbates at lower pressures.

Both the calculated selectivity values and the trend of decreasing selectivity with increasing pressure are consistent with the selectivity estimates derived by Wu et al. [8] based on IAST and simulated isotherms. In Wu et al.’s work, the selectivity estimates at low pressures align with our findings. Moreover, they predicted a continued decrease in selectivity with increasing pressure, up to approximately 80 bar. This behavior in selectivity suggests a weaker adsorption affinity for ethylene. We previously elucidated this behavior with reference to adsorption enthalpy, and in the subsequent section, we will further elaborate on it by delving into the interactions between adsorbate molecules and the ZIF-8 framework.

Another noteworthy aspect of ZIF-8’s ethane selectivity behavior is its limited dependence on temperature, as depicted in Figure 7. It is important to highlight that the maximum temperature difference between the experiments was 52 K, which had a significant impact on the quantity adsorbed (Figure 5). However, the effect of temperature exhibited remarkable similarity for both adsorbates, resulting in the selectivity remaining essentially unchanged.

From a microscopic perspective, the selectivity behavior can be comprehended by considering the thermal evolution of the ZIF-8 framework, as previously elucidated by Park et al. [29]. As the temperature rose, the affinity interactions between molecules adsorbed within the pores, including coordination bonds and dispersion forces, may have experienced a slight weakening due to thermal agitation and the heightened kinetic energy of the molecules. This reduction in interactions, driven by the increased thermal energy [29,42,43], was analogous for both ethane and ethylene, resulting in the selectivity between them remaining virtually unchanged.

It has been observed that ZIF-8 presented a high adsorption capacity for ethane and ethylene; however, it is important to consider that the thermal expansion of these gases could negatively affect their adsorption on the material. In order to minimize these effects, it was essential to control both temperature and pressure during the adsorption process. This would ensure that the porous structure of the ZIF-8 was not deformed or broken, maintaining its adsorption capacity.

### 3.3. Theoretical Results

**Figure 8:**
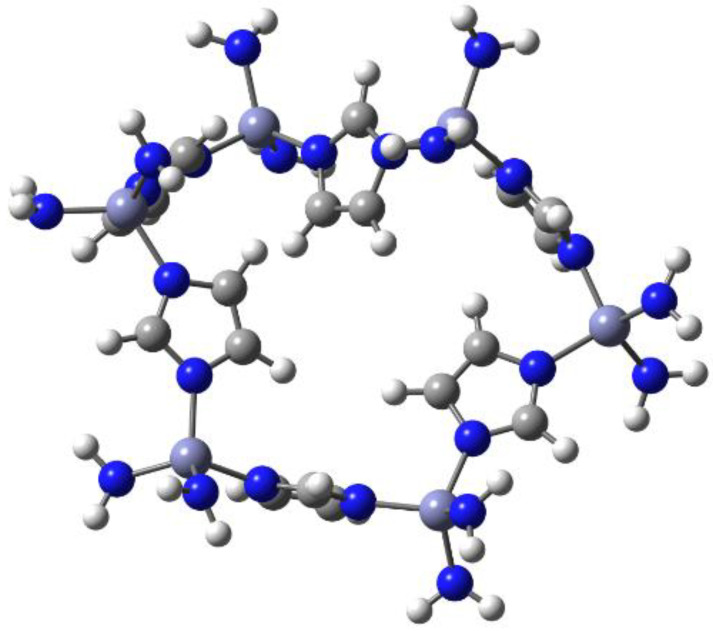
Single cell from the ZIF-8 MOF. The gray, strong blue, white, and light blue spheres represent C, N, H, and Zn atoms, respectively.

The interaction between the ZIF-8 derivative and the ethane molecules was investigated from a theoretical point of view. The modeling of the whole ZIF-8 complex can be a very difficult task and even worse, the optimization of all the geometry by a quantum chemical method; therefore, in order to have a realistic participant in the simulation of the reaction, the design of a fragment of the whole MOF was carried out (Figure 8).

Theoretical calculations were carried out putting ethane and ethylene units in the vicinity of several regions of this cell. Almost all the results were that the hydrocarbon molecules remained the same and there were no significant interactions; indeed, all the simulations that involved ethylene concluded with the separation of these molecules and the cell. However, there is a very interesting case which involved the ethane molecule.

**Figure 9:**
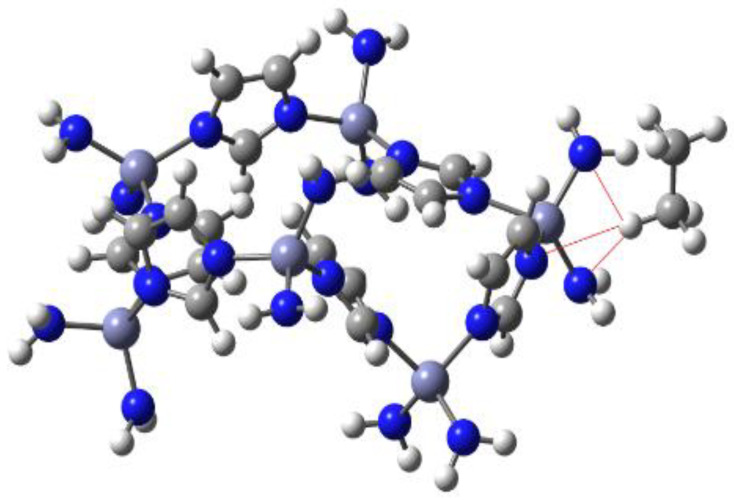
Hydrogen bonds among ethane terminal hydrogens and the MOF’s cell. The gray, strong blue, white, and light blue spheres represent C, N, H, and Zn atoms, respectively.

For individual ethane molecules near the region formed by the imidazole group and the zinc atom containing its substituents, the ethane molecules remained near the surface of the cell. The distances between the hydrogen atoms of ethane and three nitrogen atoms from the cell were 3.31 and 2.89 Å, respectively, considering the amine nitrogen atoms, and 2.59 Å between the hydrogen atoms and the nitrogen from the imidazole ring. These values suggest the strong possibility of having hydrogen bonds of a large magnitude. The indicated interactions were drawn and can be appreciated in Figure 9.

This interaction was evaluated by taking advantage of Grimme’s empirical dispersion corrections. New calculations considering this software were carried out, with the result showing extra energy with a value of 18.26 kcal/mol, which can be attributed to the new hydrogen bonds.

These results suggest that the peculiar selectivity found in this procedure has its origins in the formation of hydrogen bonds from ethane and the MOF’s surface. It seems this possibility was disabled in the case of ethylene and the reason for this could be the presence of the π bonds which precluded a good interaction of the ethylene hydrogens with the surface.

**Figure 10:**
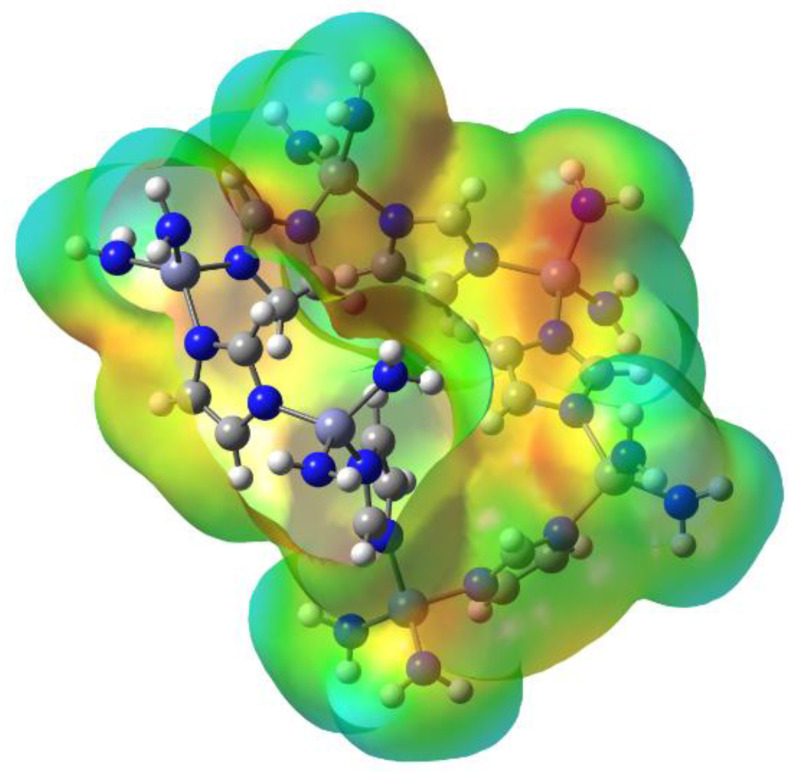
Molecular electrostatic potential mapped onto electron density from the cell of ZIF-8.

The molecular electrostatic potential mapped onto the electron density of the cell shows there was a negative charge region near the triangle formed by the three nitrogen atoms; this zone could be a trap for capturing terminal hydrogen atoms. The corresponding scheme is shown in Figure 10.

## 4. Conclusions

The isosteric enthalpy of ethane adsorption remained relatively constant at approximately −10 kJ/mol, whereas that of ethylene decreased from −4 kJ/mol to values similar to those of ethane as the loading increased.

ZIF-8 ethane selectivity decreased from 2 to 2.8 when the pressure increased up to 0.30 bar and was almost independent of temperature from 311 K to 363 K.

Theoretical calculations suggested the formation of hydrogen bonds between ethane and ZIF-8’s imidazole groups. These bonds play a crucial role in ZIF-8 ethane selectivity. The validity of this interaction was further corroborated by empirical Grimme’s dispersion corrections, which revealed an additional energy contribution of 18.26 kcal/mol attributable to the newly formed hydrogen bonds.

These findings offer a deeper insight into the interactions between ZIF-8 and hydrocarbon molecules, potentially holding significant implications for the development of efficient gas separation and storage technologies. It is advisable that future research concentrates on kinetic selectivity and mass transfer kinetics to accurately assess the practical performance of the process. Furthermore, the material’s stability in the presence of water and common flue gas impurities should be investigated to facilitate the realistic application of ZIF-8 in large-scale gas separation processes. These supplementary studies will yield a more comprehensive understanding of the applicability and practical efficiency of processes based on ZIF-8.

## Data Availability

The raw data are available upon request to the authors.

## Supplementary Materials

The following supporting information can be downloaded at: https://www.mdpi.com/article/10.3390/ma16196587/s1, Figure S1: ZLC curves for ethane and ethylene in a ZIF-8 sample at various temperatures, with a constant purge flow rate of 10 mL/min; Figure S2: Comparison between ethane and ethylene isotherms previously reported at 298 K [7] and those obtained in this work by ZLC at 311 K; Table S1: Thermodynamic properties of ethane and ethylene [36,37,38,39].
