# Supplementary material for: A Route to Understanding the Ethane Adsorption Selectivity of the Zeolitic Imidazolate Framework-8 in Ethane–Ethylene Mixtures
Source: Materials (Basel). 2023 Oct 7;16(19):6587. doi: 10.3390/ma16196587 (PMC10574225; doi:10.3390/ma16196587)
Supplement: Supplementary file 1 [file materials-16-06587-s001.zip › materials-2627150-supplementary.pdf]

Support information
A route to understand the ethane adsorption selectivity of zeo-litic imidazolate framework-8 in ethane-ethylene mixtures

Jaquebet Vargas-Bustamante, Roberto Salcedo and Jorge Balmaseda

Departamento de polímeros, Instituto de Investigaciones en Materiales, Universidad Nacional Autónoma de México. Circuito exterior s/n. Ciudad Universitaria. Coyoacán 04510. Ciudad de México. México.

S.1 Zero length column chromatography

For all ZLC experiments, the long-term response model was applied, and the average residual error function (ARE) was used. This function is defined as:

$$\mathrm{ARE} = \frac{100}{S} \sum_{K-1}^{S} \left| \frac{\left({C}/{C_0}\right)\text{calculated} - \left({C}/{C_0}\right)\text{experimental}}{\left({C}/{C_0}\right)\text{ calculated}} \right| \quad \textbf{(S 1)}$$

donde S, es el número de puntos experimentales

$$\mathrm{SOR} = \frac{100}{S} \sum_{K-1}^{S} (q \text{ experimental} - q^{*}\text{calculated})^2 \quad \textbf{(S 2)}$$

Best-fit parameters for the isotherms were found by minimizing the squared objective function of the residuals (SOR).

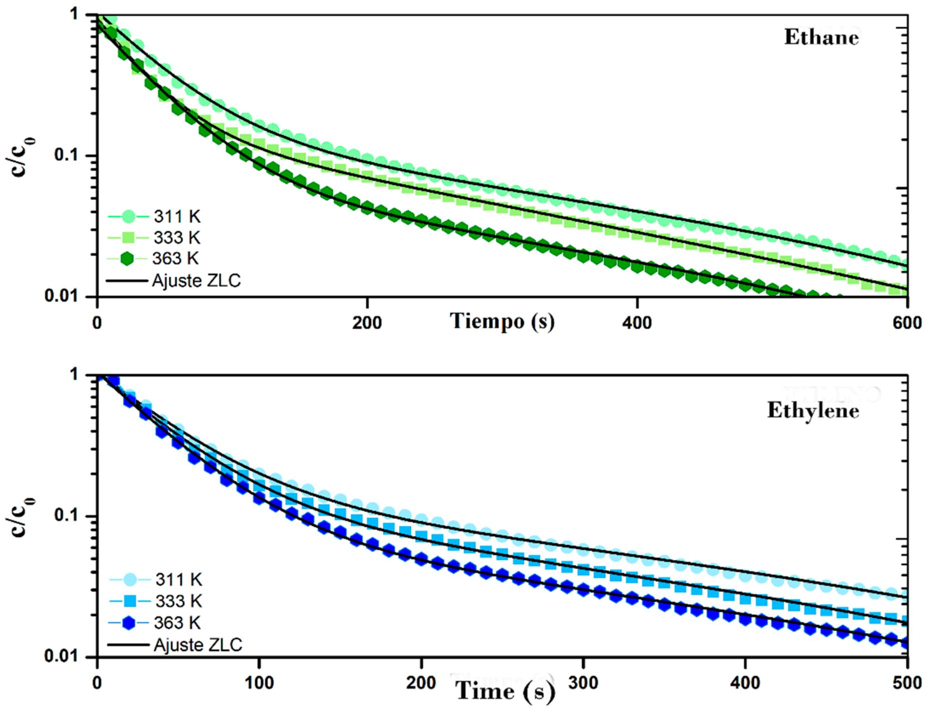

Figure S 1. ZLC curves for ethane and ethylene in a ZIF-8 sample at various temperatures, with a constant purge flow rate of 10 mL/min.

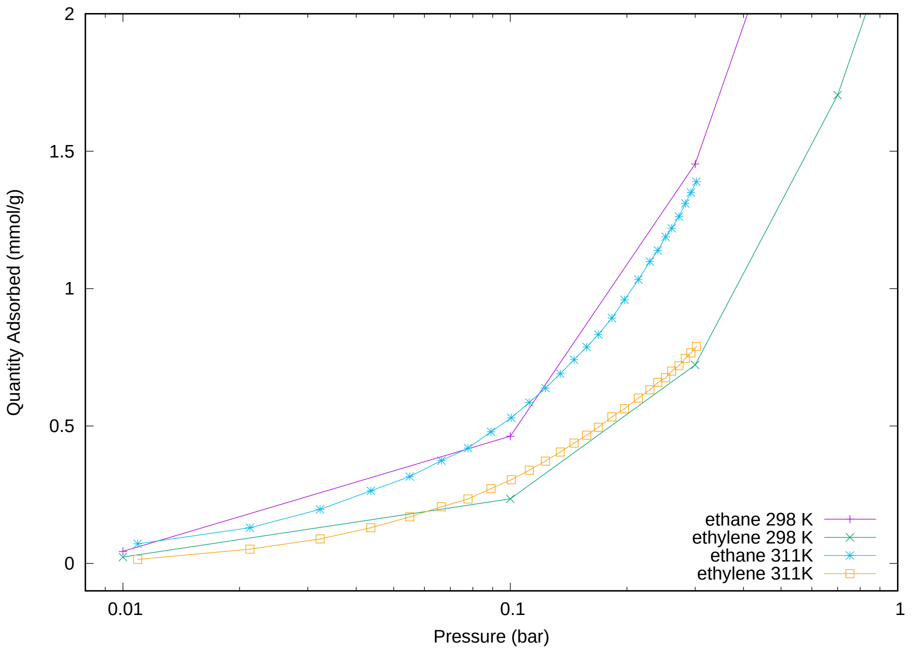

Figure S 2 Comparison between ethane and ethylene isotherms previously reported at 298 K [7] and those obtained in this work by ZLC at 311 K.

S.2 Thermodynamic properties of ethane and ethylene

Table S 1.Thermodynamic properties of ethane and ethylene.

	Ethane	Ethylene
**T_{boil} [36]**	184.6 ± 0.6 K	169 ± 0.6 K
**T_c [07]**	305.3 ± 0.3 K	282.4 ± 0.3 K
**P_c [07]**	49 ± 1bar	50.6 ± 1bar
**ρ_c [07]**	6.9 ± 0.4 mol/L	7.63 ± 0.4 mol/L
**$\Delta_{vap}H°$ [38]**	9.76 KJ/mol	13.6 KJ/mol
**Cp (gas) at 298.15 K [38]**	52.49 J/molK	42.9 J/molK
**Vapor enthalpy [39]**	15.48 kJ/mol	14.84 kJ/mol
**Vapor pressure at 293.15 K [39]**	48.2 kPa	53.3 kPa

References:

7. Bux, H., Chmelik, C., Krishna, R., Caro, J., 2011. Ethene/ethane separation by the MOF membrane ZIF-8: molecular correlation of permeation, adsorption, diffusion. J. Membr. Sci. 369,284–289.

36. Boiling Point Data" by Robert L. Brown and Stephen E. Stein in NIST Chemistry WebBook, NIST Standard Reference Database Number 69, Eds. P.J. Linstrom and W.G. Mallard, National Institute of Standards and Technology, Gaithersburg MD, 20899, https://doi.org/10.18434/T4D303, (obtenido 26 junio 2023).

37. Thermochemical Data" by Donald R. Burgess, Jr. in NIST Chemistry WebBook, NIST Standard

Reference Database Number 69, Eds. P.J. Linstrom and W.G. Mallard, National Institute of Standards and Technology, Gaithersburg MD, 20899, https://doi.org/10.18434/T4D303, (obtenido 26 junio 2023).

38. Majer, V.; Svoboda, V., Enthalpies of Vaporization of Organic Compounds: A Critical Review and Data Compilation, Blackwell Scientific Publications, Oxford, 1985, 300.

39. National Institute of Standards and Technology. (2022). Thermophysical Properties of Fluid Systems: Ethane and ethylene. Recuperado de [https://webbook.nist.gov]
